# A rapid method for detecting and distinguishing metallo-β-lactamase-and serine carbapenemase-producing *Enterobacteriales* using MALDI-TOF MS

**DOI:** 10.3389/fmicb.2022.1096987

**Published:** 2023-01-13

**Authors:** Xiaopeng Jing, Yanyan Hu, Tingting Wu, Xing Zhang, Shaofeng Luo, Wei Wang, Xiaochun Min, Ruiling Sun, Ji Zeng

**Affiliations:** ^1^Department of Clinical Laboratory, Wuhan Fourth Hospital, Wuhan, China; ^2^Clinical Microbiology Laboratory, School of Medicine, 2nd Affiliated Hospital of Zhejiang University, Zhejiang University, Hangzhou, China

**Keywords:** Carbapenemase-producing Enterobacteriaceae, MALDI-TOF MS, metallo-β-lactamases, serine carbapenemases, ethylenediaminetetraacetic acid, Phenylboronic acid

## Abstract

**Introduction:**

Carbapenemase-producing *Enterobacteriales* (CPE) are a major health threat worldwide, and therefore the development of rapid detection methods is needed. Here, we established a method to distinguish metallo-β-lactamase and serine carbapenemases using matrix-assisted laser desorption/ionization time-of-flight mass spectrometry (MALDI-TOF MS) with ethylenediaminetetraacetic acid (EDTA) and phenylboronic acid (PB).

**Methods:**

To assess the specificity and sensitivity of the method, 110 carbapenemase-producing and 72 carbapenemase-negative *Enterobacteriales* isolates were collected, among which 51 strains produced only metallo-β-lactamase, 55 strains only serine carbapenemases, and four strains both metallo-β-lactamase and serine carbapenemases. In the proposed MALDI-TOF MS method, imipenem (IPM) and the bacterial strains to be tested were mixed, EDTA and/or PB was added, and the mixture was incubated for 4 h. The carbapenemase type was confirmed by the IPM waveform spectrum before and after incubation.

**Results:**

Based on the presence, absence, and recovery of the IPM-cyano-4-hydroxy-cinnamic acid-specific waveform peak near 479 m/z, the detection sensitivity and specificity of the method were 98.2 and 100%, respectively.

**Discussion:**

Although CPE detection by MALDI-TOF MS has been studied previously, our method distinguishes between metallo-β-lactamase and serine carbapenemases, which will be very helpful for the clinical selection of antibiotics.

## Introduction

The widespread emergence and spread of carbapenem-resistant *Enterobacteriales* around the world poses problems related to inappropriate treatment of infections and has implications for infection control interventions (Gupta et al., 2011). The vast majority of CRE is caused by the production of carbapenemases (Logan and Weinstein, 2017; Wang et al., 2018). In the Ambler classification system, carbapenemases are divided into class A, B, or D based on their molecular characteristics. Class A and D carbapenemases require serine in their active sites and are also known as serine enzymes, whereas class B carbapenemases, also called metallo-β-lactamases (MBLs), require zinc for β-lactam hydrolysis (Jean et al., 2015; Bonomo, 2017; Khan et al., 2017). In *Enterobacteriales*, most class A serine carbapenemases are *Klebsiella pneumoniae* carbapenemases (KPC), whereas the most common class D serine carbapenemases are OXA-48-like. Of the transferable MBLs, imipenemase (IMP), Verona integron-encoded MBL (VIM), and New Delhi MBL (NDM) are the most common (Jean et al., 2015).

The convenient and accurate detection of carbapenemases is essential for the clinical treatment and prevention of nosocomial infections. The Clinical & Laboratory Standards Institute (CLSI) 2010 introduced the modified Hodge test method, but this method has a limited detection range and is only accurate for detecting KPCs and, thus, has been removed from CLSI recommendations (CLSI, 2010). CLSI (2012) recommended the Carba NP test method for the detection of carbapenemases in Gram-negative bacilli. However, preparation of the reagents required for this test is complicated, and the solutions cannot be stored for extended periods, limiting its clinical application (CLSI, 2012). In 2017, CLSI recommended the modified carbapenem inactivation method (mCIM; CLSI, 2017; Pierce et al., 2017). In 2018, CLSI recommended EDTA synergistic carbapenem inactivation test (eCIM) for detecting MBLs with mCIM (CLSI, 2018). In 2011, the first method to detect carbapenemase using matrix-assisted laser desorption ionization time of flight mass spectrometry (MALDI-TOF MS) was developed (Hrabák et al., 2011). It has been shown that the detection of carbapenemase activity in *Enterobacteriales* and *Pseudomonas aeruginosa* can be achieved through the detection of ertapenem, imipenem (IPM), and meropenem molecules and their natural degradation products using MALDI-TOF (Kempf et al., 2012; Hoyos-Mallecot et al., 2014; Monteferrante et al., 2016; Oviano and Bou, 2017; Sakarikou et al., 2017; Oho et al., 2021). Here, we describe the development and successful application of an MS profile generated by MALDI-TOF that utilizes the antibiotic IPM for the detection of carbapenemases and simultaneous differentiation of MBLs from serine carbapenemases in *Enterobacteriales*.

## Materials and methods

### Bacterial isolates

A total of 110 known carbapenemase-producing *Enterobacteriales* (CPE) including 57 *Klebsiella pneumoniae*, 38 *Escherichia coli*, and 15 *Enterobacter cloacae* isolates, were tested. Meanwhile, 72 carbapenemase-negative isolates, including 30 *K. pneumoniae*, 30 *E.coli*, and 12 *E. cloacae* isolates were tested. Among these 110 carbapenemase-producing isolates were 51 MBl producers (including 30 NDM-1, 12 IMP-like, and nine VIM-1 isolates), 53 KPC-2 producers, two OXA-48 producers, and four that produced both MBLs and serine carbapenemases (Table 1). All strains were identified at the species level using MALDI-TOF MS (Microflex; Bruker Daltonics, Bremen, Germany). The carbapenemase gene of these strains was detected by PCR and sequencing as previously described (Jing et al., 2018). The minimum inhibitory concentrations (MICs) for IPM were determined using the broth dilution method. NDM-1-producing *E. coli* T-EC06 and KPC-2-producing *K. pneumoniae* ATCC1705 were used as positive controls (Wang et al., 2015). The above quality control strains were donated by Professor Rong Zhang from the Second Affiliated Hospital of Zhejiang University (Hangzhou, China).

**Table 1:** Number of isolates found to produce various carbapenemases and the MICs of IPM for bacteria.

Carbapenemase-producing isolates	Type of enzyme	MIC for IPM (μg/ml)			
		≤1	4	8	≥16
*K. pneumoniae* (57)	KPC-2 (35)	0	1	2	32
	IMP-1 (7)	0	0	1	6
	NDM-1 (5)	0	0	0	5
	VIM-1 (3)	0	0	1	2
	IMP-2 (3)	0	0	0	3
	KPC-2/NDM-1 (3)	0	0	0	3
	OXA-48 (1)	0	1	0	0
*E. coli* (38)	KPC-2 (16)	0	1	1	14
	NDM-1 (16)	0	1	2	13
	VIM-1 (2)	0	0	0	2
	IMP-1 (1)	0	0	0	1
	IMP-2 (1)	0	0	0	1
	KPC-2/NDM-1 (1)	0	0	0	1
	OXA-48 (1)	0	1	0	0
*cloacae* (15)	NDM-1 (9)	0	1	1	7
	VIM-1 (4)	0	0	0	4
	KPC-2 (2)	0	0	0	2
Non-CPE^a^ (72)	TEM (10)	10	0	0	0
	SHV (8)	8	0	0	0
	CTX-M (7)	7	0	0	0
	SHV + TEM (6)	6	0	0	0
	TEM + CTX-M (9)	9	0	0	0
	SHV + CTX-M (7)	7	0	0	0
	TEM + SHV + CTX-M (15)	15	0	0	0
	Non (10)	10	0	0	0

^a^Non-CPE means non-carbapenemase-producing.

**Figure 1:**
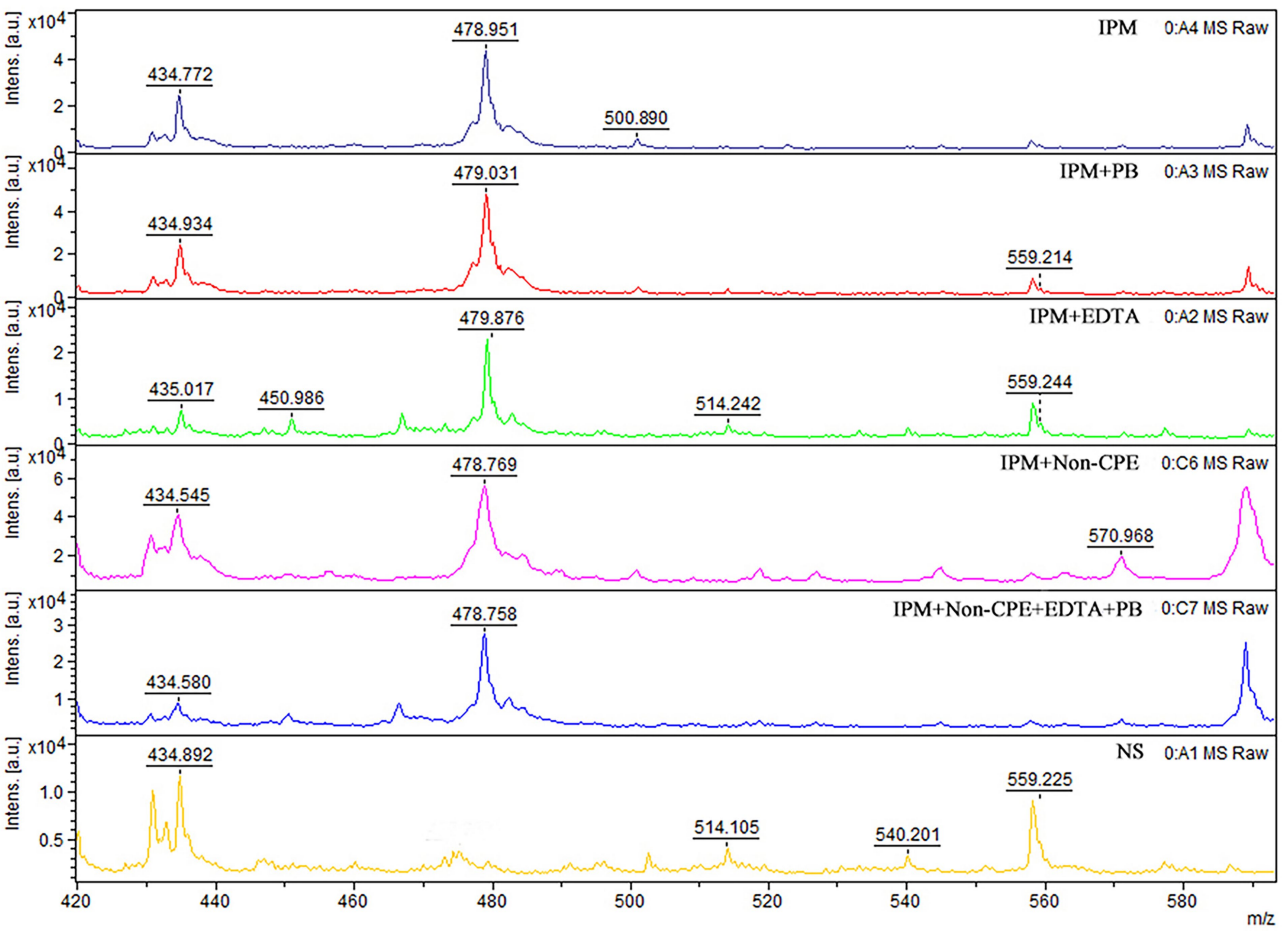
MALDI-TOF MS analysis of IPM. Mass spectra of IPM as determined using MALDI-TOF. Imipenem-cyano-4-hydroxy-cinnamic acid spectrometry peak, near 479 m/z. Non-CPE, peak exists near 479 m/z. The x axis represents mass per charge in Daltons [m/z (Da)] and the y axis represents the relative intensity. IPM, imipenem; PB, phenylboronic acid; EDTA, ethylenediaminetetraacetic acid; NS, normal saline.

*MALDI-TOF MS analysis of IPM* (Kempf et al., 2012; Oho et al., 2021) A commercially available IPM (Solarbio, Beijing, China) dissolved in 0.45% sodium chloride was used in the experiment. Then, 1 μl of the matrix (a-cyano-4-hydroxycinnamic acid, HCCA, Sigma-Aldrich, St. Louis, MO, United States) solution was mixed with 1 μl IPM, applied onto the target (Bruker Daltonics), and allowed to dry at room temperature. Mass spectra were acquired using a mass spectrometer and flexControl 3.0 software (Bruker Daltonics) operating in positive reflection ion mode. The mass range was 0–1000 m/z, and each spot was measured in duplicate in Linear Positive Mode using a 60 Hz nitrogen laser. Flex Analysis V.3.4 (Bruker Daltonics) was used for spectrum analysis, and the presence or absence of carbapenemase production was determined by the appearance of a waveform at 479 m/z representing the IPM + HCCA peak (Figure 1).

### IPM hydrolysis assay

The *Enterobacteriales* strains were cultivated in blood agar medium in an aerobic environment for 18–24 h at 35 ± 2°C. Then, a 1-μL loopful of cultured bacteria was collected and emulsified in 1 ml 0.45% NaCl as previously described by Burckhardt and Zimmermann (2011). IPM was added to a concentration of 0.25 mg/ml and incubated for 4 h at 35 ± 2°C. The mixtures were then centrifuged for 3 min at 12000 × g, and 1 μl of the supernatant was applied to each target spot, mixed with 1 μl of matrix solution, and left to dry at room temperature (Kempf et al., 2012). Two-point detection was performed for each clinical strain.

### Detection of MBLs and serine carbapenemases by MALDI-TOF MS with EDTA and PB

Briefly, four tubes were prepared for each test strain: one containing 50 μl 0.1 M ethylenediaminetetraacetic acid (EDTA), one containing 50 μl 40 mg/ml phenylboronic acid (PB), one containing both EDTA and PB, and one to which no additions were made. The remaining steps were exactly as described for the IPM hydrolysis assay.

**Figure 2:**
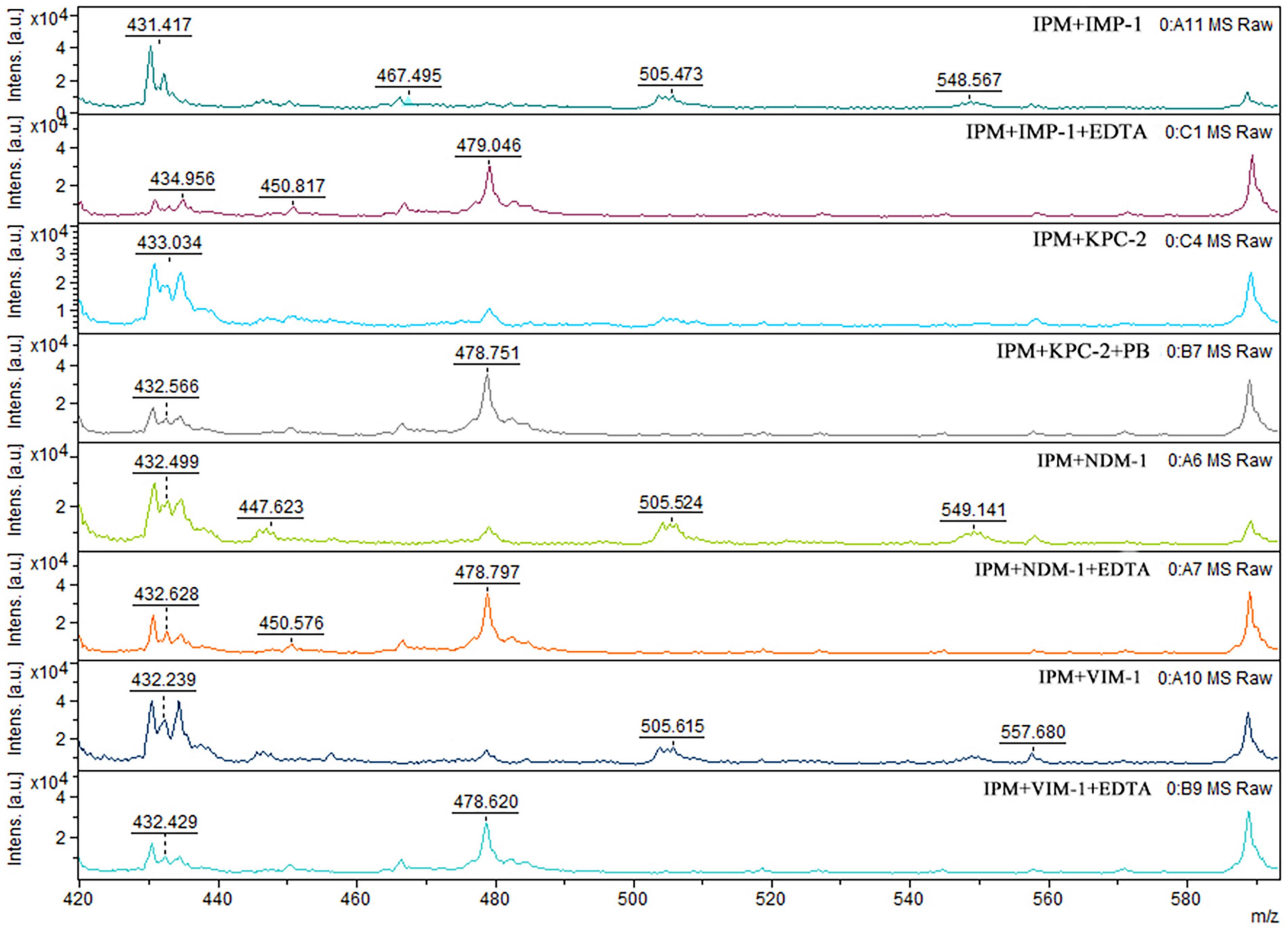
Mass spectra of the IPM hydrolysis assay with CPE. After CPE hydrolysis, the MS peak near 479 m/z disappeared. Upon addition of EDTA or PB, the MS peak near 479 m/z reappeared.

**Figure 3:**
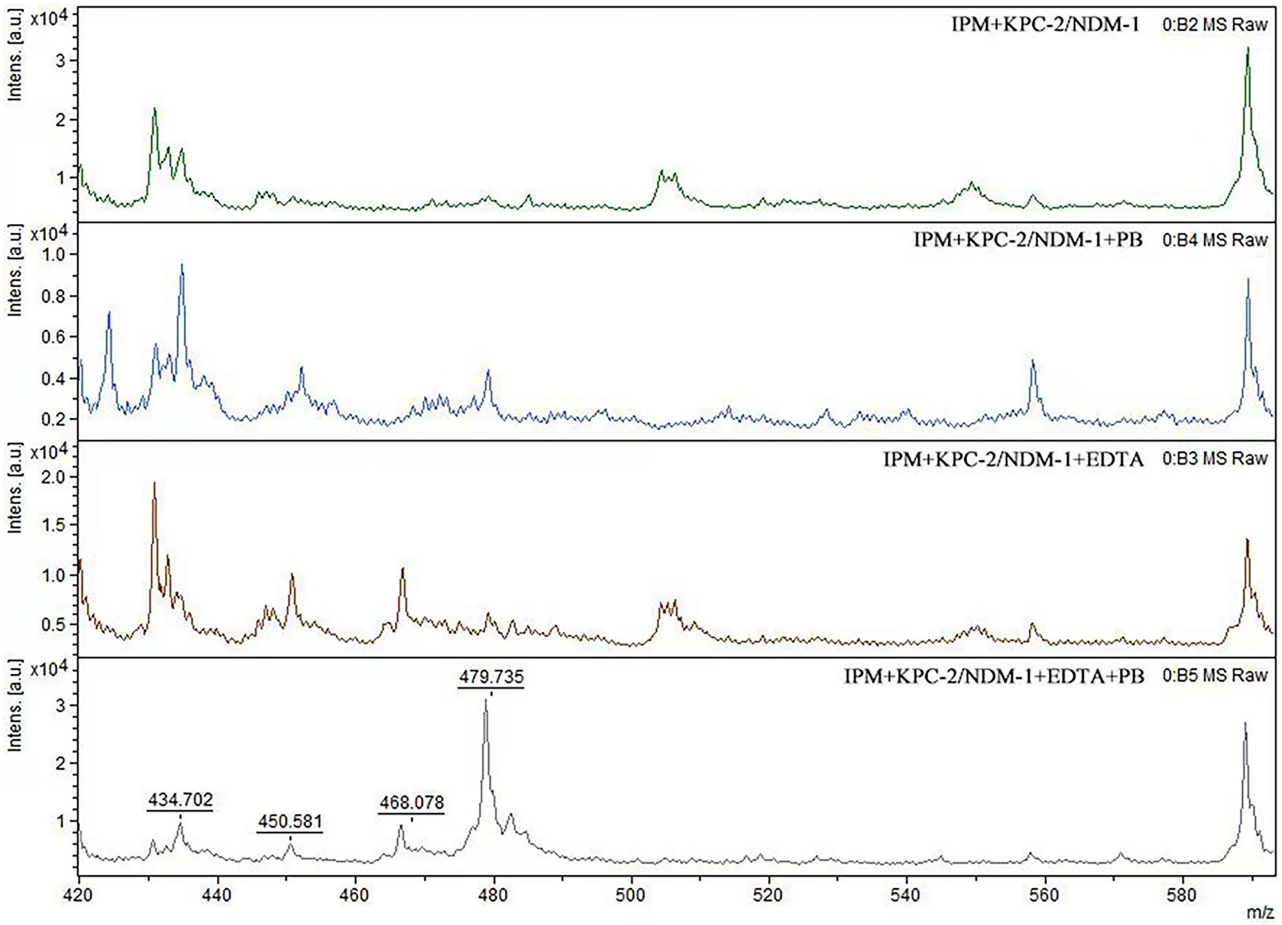
Mass spectra of the IPM hydrolysis analysis assay with dual-enzyme producing CPE. When EDTA and PB were added to MBL-and serine carbapenemase-producing strains, the 479 m/z mass spectrum peak reappeared.

When IPM is not hydrolyzed by carbapenemase, a specific IPM waveform peak appears near 479 m/z. However, this specific waveform is not observed for CPE, as IPM is hydrolyzed by carbapenemases. In this study, cases where the waveform peak near 479 m/z was absent were considered carbapenemase-positive, and those showing the waveform were considered carbapenemase-negative. When the waveform peak near 479 m/z reappeared after the addition of EDTA, the strain was considered to be MBL-producing. When the waveform peak near 479 m/z reappeared after the addition of PB, the strain was considered to be serine carbapenemase-producing. When a waveform peak did not appear after the addition of only one solution but reappeared after the simultaneous addition of the two solutions, the strain was considered to produce both enzymes (Figures 2, 3).

## Results

The MICs of all 110 CPE strains to imipenem were ≥ 4 μg/ml, and 87.3% of the isolates had an MIC ≥16 μg/ml (Table 1).

Results of IPM hydrolysis assays showed that the specific IPM waveform near 479 m/z was absent for 108 of the 110 CPE strains, whereas all 72 non-CPE strains retained the specific IPM waveform (Table 2).

**Table 2:** CPE classification as detected by MALDI-TOF with IPM, EDTA, and PB.

Genotype (*n*)	IPM: near 479 m/z^a^		adding EDTA^c^ IPM: near 479 m/z		adding PB^d^ IPM: near 479 m/z		adding EDTA and PB IPM: near 479 m/z	
	Peak appearance	No peak appears	Peak appearance	No peak appears	Peak appearance	No peak appears	Peak appearance	No peak appears
KPC-2 (53)	0	53	0	53	53	0	53	0
NDM-1 (30)	0	30	30	0	0	30	30	0
VIM-1 (9)	0	9	9	0	0	9	9	0
IMP-1 (8)	0	8	8	0	0	8	8	0
IMP-2 (4)	0	4	4	0	0	4	4	0
KPC-2/NDM-1 (4)	0	4	0	4	0	4	4	0
OXA-48 (2)	2	0	2	0	2	0	2	0
Non-CPE (72)^b^	72	0	72	0	72	0	72	0

a, CPE hydrolyzes IPM, no peak appears. b, Non-CPE does not hydrolyze IPM, peak appears. c, EDTA inhibits metalloenzymes, peak reappears. d, PB inhibits serinase, peak reappears.

For all 51 MBl-producing strains, the characteristic 479 m/z IPM peak reappeared with the addition of EDTA. The same result was observed for the 53 KPC-2-producing strains after the addition of PB. For the four MBL-and serine carbapenemase-producing strains, the characteristic 479 m/z IPM peak also reappeared with the addition of EDTA and PB. Disappearance of the characteristic IPM peak in the two OXA-48-producing strains was incomplete. The overall sensitivity and specificity for the MBLs and serine carbapenemases tested in the 110 CPE strains were 98.2 and 100%, respectively (Table 2).

In MBL-producing strains, the characteristic 479 m/z IPM peak did not reappear after the addition of PB. The IPM peak also did not reappear after EDTA was added to serine carbapenemase-producing strains. For strains producing both MBLs and serine carbapenemases, the addition of only EDTA or PB did not cause the 479 m/z peak to reappear. This suggests that EDTA and PB themselves do not affect the characteristic IPM peak (Table 2).

For both the NDM-1-producing quality control isolate *E. coli* T-EC06 and the KPC-2-producing quality control isolate *K. pneumoniae* ATCC 1705, which were used as positive controls, the characteristic IPM peak reappeared after the addition of EDTA and PB, respectively.

## Discussion

Antibiotic resistant CPE is a life-threatening disease with a 26–44% higher mortality rate than infections caused by carbapenem-sensitive bacteria and a global detection rate that is rising every year (Nordmann et al., 2011; Jeon et al., 2015; Oho et al., 2021). Therefore, timely diagnosis is essential to improve patient outcomes, choose optimal antibiotic treatments, and implement a surveillance network.

The MALDI-TOF technique is a rapid and reliable identification method that can be used for routine applications in diagnostic laboratories (Murray, 2010). This rapid, simple, inexpensive, and high-throughput proteomic system has been shown to be an effective method for both bacterial and fungal identification (van Veen et al., 2010). Recently, some method for rapid detection of CPE using MALDI-TOF were reported (Kempf et al., 2012; Hoyos-Mallecot et al., 2014; Monteferrante et al., 2016; Oviano and Bou, 2017; Sakarikou et al., 2017; Neonakis and Spandidos, 2019; Oho et al., 2021); however, these methods can only detect whether a strain produces carbapenemase or can distinguish MBLs and serine carbapenemases, but cannot detect those bacteria producing both MBLs and serine carbapenemases. In our study, we used MALDI-TOF to detect carbapenemases and distinguish between MBLs and serine carbapenemases using EDTA and PB. The underlying principle of the method is based on the fact that MBL activity is inhibited in the presence of EDTA, and serine carbapenemase activity is inhibited in the presence of PB. Thus, these antibiotics are not hydrolyzed as efficiently in the presence of EDTA and PB (Tsakris et al., 2008; CLSI, 2018).

Interestingly, carbapenemases can hydrolyze both IPM and meropenem. However, we chose to use IPM without meropenem based on the fact that the rate of enzymatic hydrolysis of IPM is much higher than that of meropenem (Queenan and Bush, 2007). Because antibiotics with a fast hydrolysis rate can increase the sensitivity of the method when the total amount of enzyme produced is low, we chose to use IPM for our experiments. Of course, there are very few strains that can only hydrolyze meropenem but not imipenem. If such strains are detected, false negative results may be caused.

At the same volume, higher concentrations of EDTA result in the chelation of more zinc ions. The antimicrobial mechanism of EDTA occurs *via* the chelation of divalent metal ions such as Ca^2+^ and Mg^2+^, which are necessary for cellular replication and growth as well as stability and replication of the outer layers of the bacterial cell wall. Therefore, in some cases high concentrations of EDTA can destabilize and remove the outer lipopolysaccharide layer, and excessive EDTA can lead to false positive results (Fukada and Ozaki, 2007). Therefore, it is important that the appropriate concentration of EDTA be used. In preliminary experiments, we tested three final concentrations of EDTA: 2.5, 5, and 10 mM (data not shown). We found that 2.5 mM EDTA resulted in negative results for some MBL-producing bacteria. However, 10 mM EDTA resulted in false positive results for a few serine carbapenemase-producing strains. At a concentration of 5 mM EDTA, no false positives or false negatives occurred. Based on these results, we selected a concentration of 5 mM EDTA.

Two OXA-48-producing strains could not be detected, and the disappearance of the characteristic IPM peak was incomplete. This may be because OXA-48 hydrolysis is weak and fails to fully hydrolyze IPM. Therefore, this method may not be suitable for detecting OXA-48-producing strains.

Although there have been many studies detecting CPE using MALDI-TOF, this method still has some deficiencies. For example, differences in antibiotic MS peaks occur due to the different matrices and buffers used by different researchers. Thus, the application of MALDI-TOF MS for pathogen resistance analysis still needs to address issues of inoculum size, incubation time, antibiotic concentration, and interpretation in order to provide rapid and reliable results.

## Conclusion

In conclusion, we report a novel approach to rapidly detect MBLs and serine carbapenemases in CPE using MALDI-TOF, a commercially available IPM, EDTA, and PB. Our study demonstrates that this assay can be routinely used in clinical microbiology laboratories. The accurate classification and detection of CPE will aid clinical drug use while preventing nosocomial outbreaks and the spread of uncontrolled superbugs.

### ICMJE statement

All authors meet the ICMJE authorship criteria.

## Data availability statement

The original contributions presented in the study are included in the article/supplementary material, further inquiries can be directed to the corresponding author.

## Author contributions

XJ, TW, and XZ isolated bacteria and performed the laboratory measurements. SL, WW, XM, and RS isolated and identified bacteria. YH and XJ performed the data analysis and the atlas production. JZ and XJ made substantial contributions to conception and design. JZ and XJ wrote and revised the manuscript. JZ drafted the manuscript. All authors contributed to the article and approved the submitted version.

## Funding

This work was partially supported by the foundation of the Wuhan Municipal Health Commission (grant number WX19Q31 and number WX18C17); the foundation of the Joint Fund of Hubei Provincial Health Commission (grant number WJ2019H378). These funds provided the fee for the collection and identification of isolates and the publication.

## Conflict of interest

The authors declare that the research was conducted in the absence of any commercial or financial relationships that could be construed as a potential conflict of interest.

## Publisher’s note

All claims expressed in this article are solely those of the authors and do not necessarily represent those of their affiliated organizations, or those of the publisher, the editors and the reviewers. Any product that may be evaluated in this article, or claim that may be made by its manufacturer, is not guaranteed or endorsed by the publisher.
